# Pixelated Micropolarizer Array Based on Carbon Nanotube Films

**DOI:** 10.3390/nano13030391

**Published:** 2023-01-18

**Authors:** Hui Zhang, Yanji Yi, Yibin Wang, Huwang Hou, Ting Meng, Peng Zhang, Yang Zhao

**Affiliations:** 1 Chinese Academy of Sciences Key Laboratory of Mechanical Behavior and Design of Materials, University of Science and Technology of China, Hefei 230022, China; 2 Department of Precision Machinery & Precision Instrumentation, University of Science and Technology of China, Hefei 230022, China; 3 Key Laboratory of Precision Scientific Instrumentation of Anhui Higher Education Institutes, University of Science and Technology of China, Hefei 230022, China; 4 Department of Modern Mechanics, University of Science and Technology of China, Hefei 230026, China

**Keywords:** polarization-sensitive devices, polarimetry, micropolarizer array, material of polarization

## Abstract

A micropolarizer array (MPA) that can be integrated into a scientific camera is proposed as a real-time polarimeter that is capable of extracting the polarization parameters. The MPA is based on highly aligned carbon nanotube (CNT) films inspired by their typical anisotropy and selectivity for light propagation over a wide spectral range. The MPA contains a dual-tier CNT pixel plane with 0° and 45° orientations. The thickness of the dual-tier structure of the CNT-based MPA is limited to less than 2 μm with a pixel size of 7.45 μm × 7.45 μm. The degree of polarization of the CNT-MPA reached 93% at a 632 nm wavelength. The specific designs in structure and semiconductor fabrication procedures are described. Compared with customary MPAs, CNT-based MPA holds great potential in decreasing the cross-talk risk associated with lower film thickness and can be extended to a wide spectral range.

## 1. Introduction

Polarization detection plays an irreplaceable role in imaging systems for containing not only geometric information but also the physical characteristics of the targets [1]. These inherent properties facilitate the widespread applications of polarimeters in passive target detection [2,3,4], navigation [5], computer vision [6], biomedicine [7], etc. Conventional polarimeters are based on time-domain or space-domain multiplexed measurements, which are accompanied by a reduction in temporary frame rate or spatial resolution [8]. An MPA integrated over a focal plane array was put forward, which addressed these issues appropriately for extracting almost full polarimetric parameters of light in real-time and parallel spaces [9]. Since then, multiple materials and designs of MPAs have been proposed over the past decades [10].

Popular anisotropic materials for MPAs are essentially focused on three categories: metal wires, dichroic molecules in polymer materials, and birefringent anisotropic crystals. Many well-performing polarized light modulators have been produced based on metal wires [4,11]. However, the wire pitch needs to be much smaller than the incident wavelength (less than 10%), which makes it difficult to fabricate for the regions below the near-infrared spectrum. Although advanced lithography techniques such as electron beam lithography or holographic lithography can be applied, high expenses still severely limit their development. A more cost-effective MPA is based on dichroic molecular absorption in stretched polymers, as in iodide-doped polyvinyl alcohol (PVA) films [8,9]. However, it is difficult to reduce the thickness of PVA-based MPAs below 10 μm. The excessive thickness causes high cross-talk risk and small tolerance to incident light angles. An MPA based on a liquid crystal [12] can reach a thickness of 0.95 μm but is limited to the visible spectrum range.

In response to the cost-effectiveness and large-scale production demands of MPAs, advanced materials and innovative techniques are under persistent efforts. Carbon nanotubes (CNTs) are typical anisotropic materials that have strong selectivity for partially polarized light propagation [13,14]. Their ultra-small diameter induces quantized wave vectors in the circumferential direction [15,16]. The discretized energy levels significantly suppress the inter-band absorption of light polarized perpendicular to the axial of CNTs [17,18]. Hence, when a light beam passes through a super-aligned CNT film, the photons with polarization perpendicular to the alignment direction of the CNTs pass through, whereas those parallel ones are absorbed [19]. The nanoscale diameter of the individual tube (10~20 nm) enables CNT-based polarimeters to work effectively over a wide spectral range. For the ultraviolet region, even the wavelengths in the regions of tens of nanometers, Kaili Jiang et al. [14] reported the remarkable polarimetric capacity of the CNT polarizer, and a 92% degree of polarization (DOP) was obtained at 325 nm wavelength. Moonyoung Jun et al. [20] prepared a CNT polarizer with a transmittance of 60% and a DOP of 70% in the visible spectral range. More significantly, in a broader terahertz range from 0.1~2.0 THz, the extinction ratio of the CNT polarizer was reported to reach nearly 37 dB [21]. The remarkable compatibility of self-assembled CNT films and semiconductor processes was reported by Hayamizu Y. et al. [22] and Fan S. et al. [23], which demonstrated the scalable fabrication of CNT devices. Our previous study [24] demonstrated that for capillary force-assembled CNT films, the optimum thickness of the film is approximately 0.7~0.8 μm (density 0.59 g/cm^3^) corresponding to the incident light of 632 nm wavelength. With optimized growth conditions and a purification process, the film achieved a transmittance of 45% and a DOP of 99%.

In this paper, a dual-tier CNT-MPA with 0° and 45° orientations is designed to extract Stokes parameters that can describe the polarization information. The 7.45 μm × 7.45 μm pixel-sized MPA is fabricated under standard MEMS technology. Subsequently, the structure and optical properties of the CNT-MPA are characterized, showing a functional layer with a thickness of less than 2 μm and DOP values of 93% and 77% for the two CNT pixel tiers at 632 nm wavelength, respectively. This work demonstrates a promising technique for the cost-effectiveness and large-scale production of nanotube-based MPAs. More significantly, the ultrafine structure of CNT films enables their applications in various spectrum ranges, as has been proved previously. The tested MPA is designed for the visible spectrum range, specifically 632 nm. Hence, the growth parameters of the CNT arrays and the selection of substrate are based on the specified wavelength, and the optical characterizations of the device are conducted under the same wavelength. To further demonstrate the versatility of the CNT-based MPA over a broader wavelength range, the DOPs at 532 nm and 808 nm are also presented. Nevertheless, the technique can be easily extended to other spectrum ranges with optimized film thicknesses and appropriate substrates for the given wavelength (e. g., silicon or germanium for the infrared range).

## 2. Structural Designs of MPA Based on Stokes Parameters

The traditional MPA consists of polarization filters in four directions, which are 0°, 45°, 90°, and 135°, respectively. However, the four-angle configuration complicates the fabrication process, and the composition thickness of the device could facilitate the crosstalk between pixels. In the case that phase information can be ignored, the polarization state of light can also be achieved by measuring two linear polarization components (nonorthogonal) along with the total intensity of the incidence [8]. The polarization states are usually expressed with Stokes vectors [25]. There are multiple expressions of the Stokes parameters, and a general form can be presented as follows (a detailed description can be found in Supplementary Document S1):

(1) $$S_{0}=\varepsilon I_{t}$$

(2) $$S_{1}=2I\left(0{}^{\circ},\;0\right)-\varepsilon I_{t}$$

(3) $$S_{2}=\frac{2[I\left(\theta ,\;0\right)-I\left(0{}^{\circ},\;0\right)\cos2\theta -\varepsilon I_{t}\sin^{2}\theta ]}{\sin2\theta }$$

where *I*_t_ represents the total intensity of the incident light, and *I* (0°, 0) and *I* (θ, 0) represent the intensity filtered by two nonorthogonal polarizers oriented at 0 and θ degrees without phase compensation, respectively. And ε is a transmittance coefficient of the transparent regions, which can be regulated by a layer of mutual attenuation film. For general target recognition in nature, where the phase information between components is not available, the three Stokes parameters *S*_0_, *S*_1_, and *S*_2_ are enough to describe the polarization information. Thus, an array design composed of a 0° CNT filter, a 45° CNT filter, and transparent regions is adopted so that the thickness of CNT-MPA can be significantly reduced.

**Figure 1:**
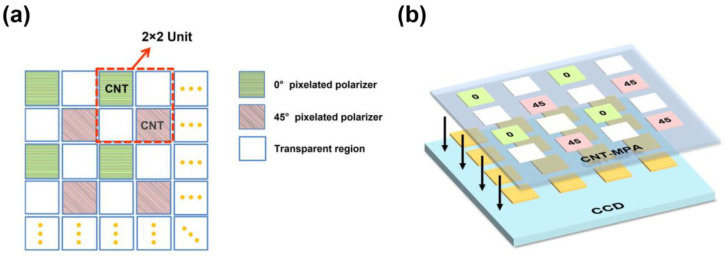
(**a**) The schematic diagram of MPA, a 2 × 2 unit containing a 0° pixelated polarizer, a 45° pixelated polarizer, and two transparent regions. (**b**) Schematic diagram of the integration of the MPA and the image sensor.

Figure 1a illustrates the dual-tier MPA design. The size of a single CNT square is 6.45 μm by 6.45 μm with a 7.45 μm pixel pitch aiming for integration with a commercial image sensor. There is a 1 μm reserved gap between adjacent pixels to avoid pixel overlaps in the fabrication process. Four pixelated areas form a 2 × 2 unit (in red dotted frame) composed of a 0° pixelated polarizer, a 45° pixelated polarizer, and two transparent regions. The unit measures *I*_t_, *I* (0°, 0)*,* and *I* (45°, 0)*,* from which the Stokes parameters can be obtained for linearly partial polarized incidence [8]. To realize polarization imaging, the MPA should be integrated into the image sensor, as shown in Figure 1b. Each pixel in the MPA should be matched with the pixel in the image sensor one-to-one and slightly larger to cover the photosensitive cell. And the integration gap will be within 1~2 μm. Since the fill factor of MPA is not 100%, there will be diffraction between the MPA and the image sensor, which introduces cross-talk. Based on Kirchhoff’s diffraction theory [1], the light intensity diffracted to the boundary of the adjacent pixel is only 3~5% of the light intensity of the corresponding pixel center, which is within the acceptable range.

## 3. Fabrication of a CNT-MPA Based on a Semiconductor Process

**Figure 2:**
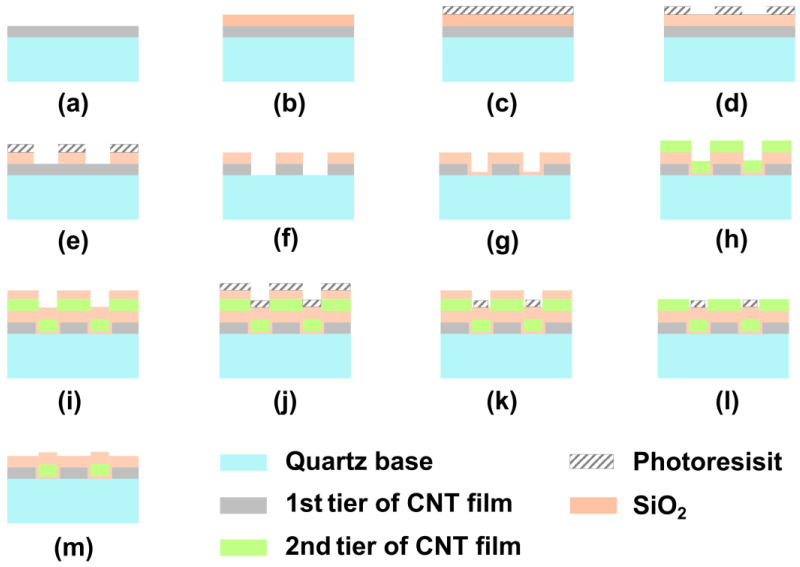
The fabrication process of a dual-tier micropolarizer array. (**a**) The first layer of CNT film is grown on the quartz substrate. (**b**) A layer of SiO_2_ is grown on the CNT film as a hard mask. (**c**,**d**) Transferring the pattern through lithography and etching process to the photoresist. (**e**) Transferring the pattern to the hard mask via selective etching. (**f**) Transferring the pattern to the first layer of CNT film. (**g**) A layer of SiO_2_ is grown as a protection layer. (**h**,**m**) The same steps of transferring the pattern to the 2nd tier of CNT film, referring to steps (**a**,**f**).

**Figure 3:**
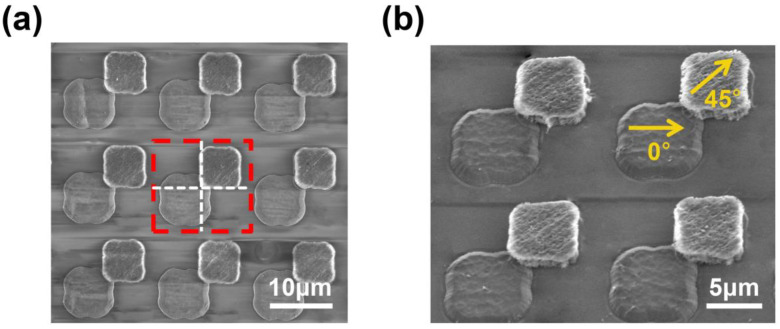
(**a**) Top view of the fabricated dual-tier MPA under SEM and the 2 × 2 unit is framed with red lines, and the two pixels are distinguished by a white cross. (**b**) The magnified SEM image of (**a**) and tilted by 30 degrees shows the height contrast and CNT orientation difference between the squares in the two layers.

The multi-walled CNTs (diameter ~20 nm) are grown by water-assistant vapor deposition using Fe film (2 nm) as the catalyst. Taking C_2_H_2_ as the carbon source, the length of CNTs can reach ~500 μm at 680 °C for 15 min. By patterning the catalyst into evenly spaced lines, the forest sheets are grown. Highly aligned CNT film is prepared by capillary zippering of vertically aligned CNT forest sheets [22]. A detailed process for the preparation of CNT films can be found in our previous work [24]. A 500 μm thick quartz wafer serves as a substrate for applications in the visible spectrum. Our previous work indicates an optimum thickness of the film is approximately 0.7~0.8 μm (density 0.59 g/cm^3^), and the corresponding line width of the catalyst for CNT forest sheets is 30 μm with a pitch of 500 μm. The following steps describe the fabrication procedures of the dual-tier MPA:

(1) The self-assembled CNT film with a thickness of ~700 nm adheres to the quartz substrate tightly due to van der Waals forces (Figure 2a). The initial orientation of the film is defined as 0 degrees. The densified CNT film is baked in the air at 400 °C for 20 min to remove the amorphous carbon and improve the transmittance of the film [26].

(2) Under plasma-enhanced chemical vapor deposition (PECVD, Plasma Pro System100, Oxford Instrument, UK), a layer of 200 nm SiO_2_ is deposited on the CNT film as a hard mask (Figure 2b);
(3) The predesigned pixelated pattern is defined through the standard lithography method (Figure 2c,d), which is described in detail in Supplementary Document S2;
(4) The hard mask is etched in reactive ion etching (RIE, Plasma Pro NGP 80, Oxford Instrument, UK) with CHF_3_ as the reaction gas (a detailed recipe can be found in Supplementary Document S2, Table S1). The preset pattern is transferred to the hard mask, as shown in Figure 2e;
(5) Oxygen was used as the etching gas (a detailed recipe can be found in Supplementary Document S2, Table S2), and the CNT film was etched in an inductively coupled plasma metal etching machine (ICP, Plasma System 100 ICP180, Oxford Instrument, UK). Under the protection of the hard mask, the predesigned pixelated pattern is transferred to the CNT film, which can be seen in Figure 2f;
(6) A layer of 200 nm PECVD SiO_2_ (a detailed recipe can be found in Supplementary Document S2, Table S3) is grown on the sample surface as a protective layer to protect the CNT arrays from the follow-up operations (Figure 2g);
(7) The second tier of self-assembled CNT film is grown directly on the protective layer (Figure 2h). The catalyst lines [24] of the second CNT film have a 45-degree angular offset from those of the first tier of CNT film. After the self-assembly process of the CNT film, the orientation of the second layer of the CNT film has a 45-degree angular offset from those in the first layer;
(8) A layer of 200 nm SiO_2_ is grown on the second tier of CNT film as a hard mask (Figure 2i). Through lithography and etching procedures similar to those described above (c–f), the predesigned pixelated pattern of the second tier of MPA is transferred to the 2nd tier of the CNT film (Figure 2j–m).

Additional steps: in the further process of integrating into an image sensor, a layer of attenuation film should be deposited on the transparent regions to guarantee the dynamic response of MPA. It can be accomplished through a film deposition via sputtering, followed by the lithography and etching process to expose the CNT areas.

## 4. Characterizations of the Dual-Tier MPA

### 4.1. The Structural Characterization of the Dual-Tier MPA

Through the steps depicted in Figure 2, a dual-tier MPA with one set in 0° polarization and another in 45° was achieved. Scanning electron microscope (SEM, FESEM 8500, Agilent, USA) images (Figure 3) reveal the arrangements of the MPA and the orientations of the two sets of polarizers. Figure 3a shows the top view of the dual-tier structure. The pixel size of the first tier seems larger than that of the 2nd tier due to the 200 nm SiO_2_ protective layer deposited in step (6) (Figure 2g). When the chip was tilted for ~30 degrees (Figure 3b), we can observe the height contrast and offset of the orientations between the two tiers. Theoretically, the height of the dual-tier structure depends on the distance from the substrate to the second layer of CNT film (3 × 200 nm SiO_2_ and ~700 nm CNT film), which is around 1.3 μm. However, there is a bit of overlap between two tiers of CNT pixels due to misalignment, which can be observed in Figure 3b. Therefore, the total height of the dual-tier MAP is a little bit more than expected but less than 2 μm. Since there is a reserved gap of 1 μm between the adjacent pixels, the overlap between the 0° and 45° corresponding pixels, caused by the low accuracy of the mask aligner (2 μm), is less than 5% of the whole pixel area. As the optical performance is determined by the entire pixel area, the crosstalk generated from the overlap is acceptable. Furthermore, this misalignment can be avoided by applying higher-precision lithography equipment.

### 4.2. The Optical Properties of the Dual-Tier MPA

**Figure 4:**
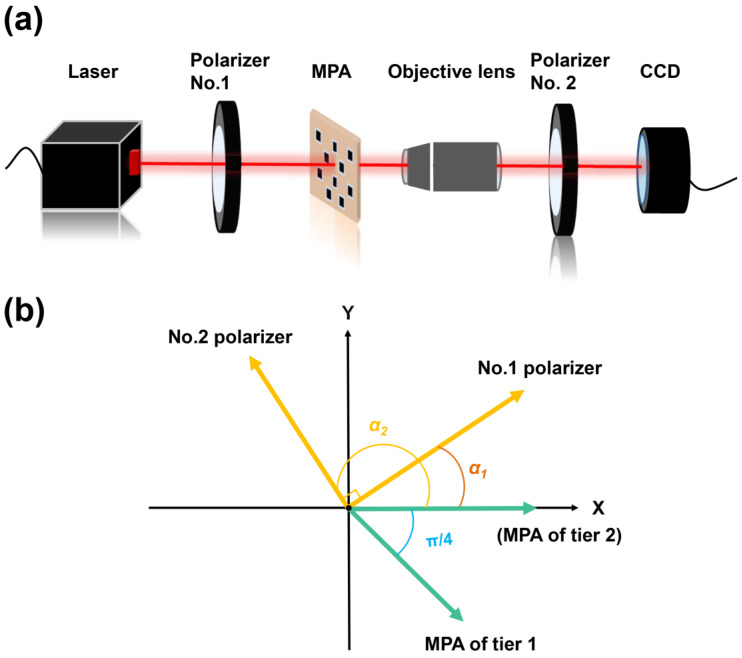
(**a**) Schematic diagram of the dark-field measuring system for MPA polarization property measurement. (**b**) The relative positions of the polarizing angle of MPA and polarizers in the same coordinate.

Since the transparent region of the MPA receives the total intensity while the CNT pixel receives less than 50% of the total intensity, the large intensity difference will affect the dynamic range of the sensor. Therefore, in the practical integration process, *S*_0_ should be regulated by the transmittance coefficient (ε) to a similar level as *S*_1_ and *S*_2_ by depositing a layer of attenuation film on the transparent region. Since different wavelengths correspond to different optimal CNT film thicknesses and transmittances, the ε should be optimized for the operating wavelength range. As the thin Chromium film is of good optical transparency [27,28] from the visible to the near-infrared range and is of good compatibility with the semiconductor process, in this work, for the 700 nm CNT film with a transmittance of 45% at 632 nm wavelength, the corresponding attenuation film is a thin layer of Cr of about 20 nm thick. Prior to the integration of MPA with imaging devices, the stand-alone CNT-MPA is characterized in a far-field setup. To refrain from the crosstalk among the adjacent pixels due to diffraction, a pair of orthogonal polarizers (polarizers No. 1 and No. 2) is set to convert the system to dark field measurement. The experiment apparatus is shown in Figure 4a.

The randomly polarized beam of laser (HNL050R, 632.8 nm, 5 mW, Thorlabs, USA) is transformed into linearly polarized light by the standard polarizer No. 1 (GCL-05, Daheng Optics, China). As the degree of polarization of the laser beam is measured to be less than 10%, the random amplitude variations of linearly polarized light waves filtered by polarizer No. 1 can be ignored. The linearly polarized light is secondarily filtered by the MPA, and the image of MPA is amplified by the objective lens (×40) and captured by the image sensor. There is a second standard polarizer (No. 2) in front of the image sensor, which is orthogonal to polarizer No. 1. To ensure the orthogonality of the two commercial polarizers, the intensity of light passing through the transparent regions is regulated to almost zero by rotating the relative angles between them. Therefore, the transparent regions appear dark (*I* = 0) in the final image, while the CNT pixels show varying intensities according to their angle differences with polarizers No. 1 and No. 2. The electric filed vector of the CNT pixels (E_2_) captured by image sensor can be described as follows:

(4) $$E_{2}=G_{\mathrm{polarizer}2}\;\cdot G_{\mathrm{MPA}}\cdot E_{1}$$

where $E_{1}=E_{1}\left(\begin{array}{c} \cos\alpha_{1}\\ \sin\alpha_{1} \end{array}\right)$ is the electric field vectors of linearly polarized light filtered by polarizer No.1. Since polarizer No.2 is orthogonal to polarizer No.1, the Jones matrix for polarizer No. 2 can be expressed as:

(5) $$G_{\mathrm{polarizer}2}=\left(\begin{array}{cc} \cos^{2}(\alpha_{1}+\frac{\pi }{2}) & \frac{1}{2}\sin2(\alpha_{1}+\frac{\pi }{2})\\ \frac{1}{2}\sin2(\alpha_{1}+\frac{\pi }{2}) & \sin^{2}(\alpha_{1}+\frac{\pi }{2}) \end{array}\right)$$

The polarization angles of both tiers of the MPA are indicated in Figure 4b. Hence, the Jones matrices for both tiers of MPA can be represented as:

(6) $$G_{\mathrm{MPA}=}\left\{\begin{array}{cc} \left[\begin{array}{cc} 1/2 & -1/2\\ -1/2 & 1/2 \end{array}\right] & \;1\mathrm{st}\;\mathrm{tier}\\ \left[\begin{array}{cc} 1 & 0\\ 0 & 0 \end{array}\right] & \;2\mathrm{nd}\;\mathrm{tier}\; \end{array}\right.$$

Therefore, the electric field and corresponding intensity received by the image sensor can be obtained as follows:

(7) $$E_{2}=\left\{\begin{array}{l} \begin{array}{ll} \frac{1}{2}E_{1}\left(\begin{array}{c} \sin\alpha_{1}\cos2\alpha_{1}\\ -\cos\alpha_{1}\cos2\alpha_{1} \end{array}\right) & 1\mathrm{st}\;\mathrm{tier}\\ E_{1}\left(\begin{array}{c} \sin^{2}\alpha_{1}\cos\alpha_{1}\\ -\cos^{2}\alpha_{1}\sin\alpha_{1} \end{array}\right) & \;2\mathrm{nd}\;\mathrm{tier} \end{array} \end{array}\right.$$

(8) $$I_{2}={\left|E_{2}\right|}^{2}=\left\{\begin{array}{l} \begin{array}{ll} \frac{1}{8}[I_{1}(1+\cos4\alpha_{1}))] & 1\mathrm{st}\;\mathrm{tier}\\ \frac{1}{8}[I_{1}(1-\cos4\alpha_{1})] & 2\mathrm{nd}\;\mathrm{tier} \end{array} \end{array}\right.$$

where *I*_1_ is the intensity corresponding to the electric field E_1_. According to Equation (8), the output intensity (*I*_2_) sinusoidally changes with a period of 𝜋/2, and there is a phase offset of 45 degrees between the intensities on the first and second tiers.

**Figure 5:**
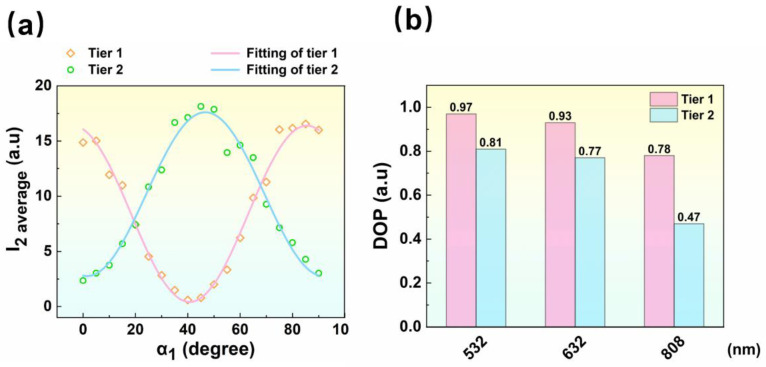
(**a**) The light intensities of CNT pixels, along with the changes in polarizing angle ($\alpha_{1}$), are depicted in tier 1 and tier 2. The fittings of both tiers are demonstrated, which follow Malus’ law and show a phase offset of ~39 degrees. (**b**) The DOP of pixels in both tiers is measured at three wavelengths of laser, which are 532 nm, 632 nm, and 808 nm.

To investigate the polarization responses of both tiers of CNT arrays, the pair of the standard polarizers (No. 1 & No. 2) rotates synchronously. The polarizing angle ($\alpha_{1}$) varied from 0 to 90 degrees with a step size of 5 degrees, and the intensity variations of the pixels in both tiers were captured by the image sensor. When $\alpha_{1}$ equals 0, the brightest illumination appears in the CNT pixels of tier 1. The output intensity *I*_2_ was averaged over each CNT pixel as the *I*_2 average_, and the variations of intensity in both tiers versus $\alpha_{1}$ are plotted in Figure 5a.

As demonstrated in Figure 5a, the average intensities of both CNT pixel tiers closely follow Malus’ cosine law, varying with the polarizing angle ($\alpha_{1}$). The phase offset between the two fitting cosine functions is approximately 39 degrees, which exhibits a 6-degree deviation from the expected phase offset (45 degrees). The deviation mainly comes from the uncertainties of the fabrication process. Although theoretically, the phase offset is not needed to be strictly 45 degrees according to Equations (1)–(3), there should be an optimal range for polarization angle θ due to the measurement uncertainties in real applications. As $S_{2}=f[I(\theta ,0),I(0^{\circ },0),\varepsilon I_{t}]$, the indirect measurement error of *S_2_* would be caused by the direct measurement error of $I(\theta ,0),I(0^{\circ },0)$, and $\varepsilon I_{t}$. Here, an error transfer function of *S_2_* can be defined as:

(9) $$H(\theta )=\sqrt{\frac{\partial f}{\partial I(\theta ,0)}\delta_{I(\theta ,0)}+\frac{\partial f}{\partial I(0^{\circ },0)}\delta_{I(0^{\circ },0)}+\frac{\partial f}{\partial (\varepsilon I_{t})}\delta_{\varepsilon }I_{t}}$$

Under the assumption that the direct measurement error $\delta_{I(\theta ,0)}$, $\delta_{I(0^{\circ },0)}$, and $\delta_{\varepsilon }I_{t}$ can be substituted by the same measurement error δ for simplifications, Equation (9) can be rewritten as:

(10) $$H(\theta )=\sqrt{4\delta (1+\cos^{2}2\theta +\sin^{4}\theta )/\sin^{2}2\theta }$$

Therefore, the optimum angle difference between the two tiers is 45°. And the 39° angle difference leads to an increase of the error transfer function H(θ) by less than 1%, which is considered within the acceptable range.

The DOP describes the polarization capability of polarimeters, expressed as (*I*_max −_ *I*_min_)/(*I*_max_ + *I*_min_), where *I*_max_ and *I*_min_ represent the maximum and minimum light intensity passing through the polarimeters. The range of DOP is 0~1, where 0 means unpolarized while 1 means perfectly polarized. To demonstrate the applications of the CNT-MPA in various wavelengths, the characterizations are subsequently carried out with another two wavelengths, green (532 nm) and near-infrared (808 nm). The DOP of pixels in both tiers for 532 nm, 632 nm, and 808 nm are listed in Figure 5b. The response of the DOP to CNT film thickness is sensitive, and the grown batches have inconsistencies. For optical multilayer structures, thickness is one of the key indicators in terms of the final performance of the device, in which the thickness of CNT film is particularly important for polarization properties. Therefore, in the previous work, we quantitatively analyzed the relationship between the thickness of CNT film and polarization capacities. Although the preparation parameters of CNT-MPA are predefined at 632 nm wavelength, the DOP exhibits best at 532 nm and poorly at 808 nm wavelength, which functions in a certain range. As shown in Figure 5b, the highest DOP of the CNT pixel approaches 0.97, which is smaller than that of commercial polarizers. However, for polarization imaging, a DOP of more than 0.9 provides polarimetric signal preservation of approximately 90%, which is within consideration [29]. The polarization capacities of the CNT film is mainly relying on the stability of the grown environment, which reaches 0.99 of DOP in our previous work at 632 nm wavelength [24]. The DOP differences between the two tiers are generated by the uncertainties in the different growth batches. The non-uniformity (NU) of MPA intrinsically exists in the nano-fabrication process. A 20% variation of optical responses may occur across the imaging array, even for metal wire MPAs [30]. Therefore, pre-calibration of NU is necessary to ensure the quality of the images. Two calibration methods (single-pixel and super-pixel) are compared by Powell and Gruev [31], and Junchao Zhang proposed a more effective method that can mitigate NU error and achieve better visual results [32].

**Figure 6:**
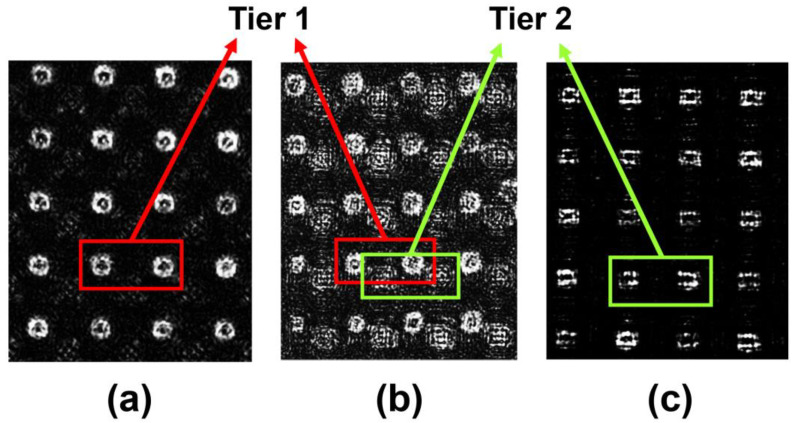
Images of the dual-tier MPA illuminated by incident polarized light at different incident angles. (**a**) 0 degrees. (**b**) 22.5 degrees. (**c**) 45 degrees.

By setting the polarizing angle ($\alpha_{1}$) as 0, 22.5, and 45 degrees, the images recording the intensity variations in MPA are visualized and presented in Figure 6. In Figure 6a, where $\alpha_{1}$ equals 0°, the CNT pixels in tier 1 show more brightness than other regions (in the red frame), while the CNT pixels in tier 2 appear opaque. As $\alpha_{1}$ increases to 22.5°, the CNT pixels in both tiers appear bright, as shown in Figure 6b (in red and green frames). Due to the existence of a height difference between the two tiers, the pixels in tier 2 seem slightly blurred and unfocused, while the system focuses on tier 1. As expected, the illuminated pixels shifted from tier 1 to tier 2 when $\alpha_{1}$ equals 45°, as shown in Figure 6c (in green frames). Hence, the imagery results roughly conform to the designed optical properties of the dual-tier MPA. There are hollow dark appearances in the pixels shown in Figure 6 due to diffraction in the far-filed detection system. As in actual use, there is only a 1~2 μm integration gap between MPA and the image sensor, which will eliminate the influence of stray light.

## 5. Conclusions

Taking full advantage of the 1-D structure of CNTs, a double-tier CNT-MPA is successfully designed and fabricated for the visible spectrum range. The characterization results indicate that CNT-MPA exhibits good polarization properties in a certain spectrum range, as high as 93% of DOP at the designed wavelength. The polarization capacities of the CNT-MPA for 532 nm and 808 nm wavelength are also inspected to demonstrate the robustness of the technique to a wide spectrum range. With a DOP comparable to metal MPAs, the fabrication procedure utilizes the standard MEMS technique, which is cost-effective and applicable for large-scale production. The considerably thin functional layer, which is less than 2 μm, significantly limits the crosstalk among pixels compared to polymer film MPAs. More importantly, profiting from the wide spectral characteristics, CNT-based MPA technology can be easily extended to much wider wavelength ranges than current technologies can cover, such as ultraviolet, infrared, and up to THz range as well. The experiment also reveals some drawbacks, such as the nonuniformity of the CNT film and the uncertainties of the fabrication process that could impose variations in DOP over different pixel tiers and also an offset of the orientation angles. Hence, future work should focus on the improvement of CNT film quality and the development of calibration and correction methods.

## Data Availability

All data generated or analyzed during this study are included in this published article (and its Supplementary Information Files).

## Supplementary Materials

The following supporting information can be downloaded at: https://www.mdpi.com/article/10.3390/nano13030391/s1, Table S1. The etching parameters of SiO_2_ via RIE. Table S2. The etching parameters of CNT film via ICP. Table S3. The deposition parameters of SiO2 via PECVD.
