# Supplementary material for: Pixelated Micropolarizer Array Based on Carbon Nanotube Films
Source: Nanomaterials (Basel). 2023 Jan 18;13(3):391. doi: 10.3390/nano13030391 (PMC9920055; doi:10.3390/nano13030391)
Supplement: Supplementary file 1 [file nanomaterials-13-00391-s001.zip › nanomaterials-2160681-supplementary.pdf]

S1. The general Stokes parameters expression

For a quasi-monochromatic light propagating in the z direction, the electric-field vector can be decomposed into x and y components, which can be written as:

$$\mathbf{E}x = a_x \exp(2\pi \mathrm{v} t - \varphi_x)$$
$$\mathbf{E}y = a_y \exp(2\pi \mathrm{v} t - \varphi_y) \qquad (1)$$

where a_x and a_y are the instantaneous electrical-field amplitudes in the x and the y directions and the corresponding phases are φ_x and φ_y. These qualities can not be detected directly, therefore, four Stokes parameters were introduced to describe the polarization state:

$$S_0 = \langle a_x^2 \rangle + \langle a_y^2 \rangle$$
$$S_1 = \langle a_x^2 \rangle - \langle a_y^2 \rangle \qquad (2)$$
$$S_2 = 2\langle a_x a_y \cos(\varphi_y - \varphi_x) \rangle$$
$$S_3 = 2\langle a_x a_y \sin(\varphi_y - \varphi_x) \rangle$$

Where $\langle \rangle$ means time average as all these quantities are time-varying. S_3 represents the circularly

polarized component that could be negligible for general target recognition in nature and $\left(\varphi_y - \varphi_x\right)$

can be regarded as 0. Take a more general case into consideration: the intensity of light passing through the polarizer with a direction of θ respect to the x-axis can be described as follows:

$$I\left(\theta,\ 0\right) = \langle a_x^2 \rangle \cos^2\theta + \langle a_y^2 \rangle \sin^2\theta + 2\langle a_x a_y \rangle \sin\theta\cos\theta \qquad (3)$$

The Stokes parameters in Eq. (2) can be transferred as follows:

$$S_0 = \langle a_x^2 \rangle + \langle a_y^2 \rangle = \varepsilon I_{\mathrm{t}}$$
$$S_1 = \langle a_x^2 \rangle - \langle a_y^2 \rangle = 2I(0°,\ 0) - \varepsilon I_{\mathrm{t}}$$
$$S_2 = 2\langle a_x a_y \cos(\varphi_y - \varphi_x) \rangle$$
$$= \frac{I\left(\theta,\ 0\right) - (\langle a_x^2 \rangle \cos^2\theta + \langle a_y^2 \rangle \sin^2\theta)}{\sin\theta\cos\theta} \qquad (4)$$
$$= \frac{I\left(\theta,\ 0\right) - [I(0°,\ 0)\cos^2\theta + (\varepsilon I_{\mathrm{t}} - I(0°,\ 0))\sin^2\theta]}{\sin\theta\cos\theta}$$
$$S_2 = \frac{2[I\left(\theta,\ 0\right) - I\left(0°,\ 0\right)\cos 2\theta - \varepsilon I_{\mathrm{t}} \sin^2\theta]}{\sin 2\theta}$$

A layer of attenuation film should be deposited on the transparent region to match the intensity in each section and avoid saturation in CCD. In that case, I_{t} in S_0 should be corrected with a coefficient ε, which can be calibrated.

S2. Detailed information on MPA fabrication in each step.

In the process of fabricating the MPA, some microelectronics equipment is applied and the corresponding recipes are set. For the reproduction of the fabrication method, details are given below:

Step 3：Photoresist AZ5214 is applied and the coating speed is over 3000 rpm. Then, bake at 100 ℃ for one minute. The exposure time of the mask aligner (Karl Suss, MABA6) is 2 s, and the light intensity is 19.5 MW/cm^2@365 nm. Before development, the wafer should be baked at 120 ℃ for 1 min and subjected to 16 seconds of floodlight exposure.
Step 4：The etching parameters of SiO_2 via RIE are listed in the table below:

Table S1. The etching parameters of SiO_2 via RIE

CHF_3	Ar	Pressure	Power	Etching rate
25 sccm	25 sccm	30 mTorr	200 W	30 nm/min

Step 5：The etching parameters of CNT film via ICP are listed in the table below:

Table S2. The etching parameters of CNT film via ICP

O_2	Pressure	Temperature	ICP power	RF power	Etching rate
1500 sccm	50 mTorr	50 ℃	1500 W	100 W	1200 nm/min

Step 6：The deposition parameters of SiO2 via PECVD are listed in the table below:

Table S3. The deposition parameters of SiO2 via PECVD

SiH_4/N_2	N_2O	Pressure	Power	Temperature
150 sccm	715 sccm	850 mTorr	200 W	300 ℃
